# Effects of dance training on core muscle strength and physical fitness in higher education: a review

**DOI:** 10.3389/fpubh.2026.1819552

**Published:** 2026-06-08

**Authors:** Zhijun Huang

**Affiliations:** Department of Dancing, Guangdong Polytechnic Normal University, Guangzhou, China

**Keywords:** balance, Core muscle strength, dance training, dance-based interventions, flexibility, physical fitness

## Abstract

Dance training places unique and intense demands on the core musculature, requiring strength, stability, and control for complex movements. This narrative review synthesises available evidence on the effects of structured dance training on core muscle strength and physical fitness in college and university students. Thirteen empirical studies, identified through searches of PubMed, Google Scholar, EBSCO, ScienceDirect, and IEEE Xplore, were included. Dance styles ranged from ballet, jazz, Zumba, and hip-hop to sport dance and classical Chinese dance, with intervention durations from 4 weeks to three semesters. A number of studies reported direct core outcomes, such as transversus abdominis activation and trunk endurance, showing improvements after dance programmes, especially when combined with supplementary core exercises. Other studies documented indirect benefits, including better hamstring flexibility, hip extensor strength, static and dynamic balance, coordination, and self-reported body awareness and emotional regulation. Overall, the evidence suggests that dance training may enhance trunk endurance, balance, flexibility, and explosive power in university students. However, the literature has notable limitations: small and predominantly female samples, heterogeneous outcome measures, frequent lack of active control groups, and few objective direct core assessments. Consequently, strong conclusions about the specific effects of dance on core muscle strength cannot yet be drawn. Dance training appears a feasible and engaging activity for university health promotion, but future research should use standardised dance protocols, direct core outcome measures, and adequately powered, diverse samples.

## Introduction

Dance is a physically demanding art form that requires exceptional core strength, stability, and control to execute complex movements with precision and grace (1). From ballet’s turned-out positions to modern dance floor work and classical Chinese dance acrobatics, the core musculature underpins virtually all dance movements (2–4). Balance, transitions, and power for jumps and turns depend on coordinated core action (5). Dance training inherently integrates core-driven movements that demand balance, coordination, and controlled force transfer (6, 7). In recent years, basic dance training has been integrated into college and university curricula to build physical competence, mental health, and injury resilience (8–10).

Physical inactivity is particularly prevalent among university students, with studies reporting that over 20% fail to meet WHO physical activity guidelines and nearly half sit for eight or more hours daily (11). This review summarises the effects of structured dance training on physical fitness, including muscular endurance, flexibility, balance, coordination, and psychological well-being, among college and university students (12). Recent study shows that dance interventions may enhance physical self-esteem and reduce anxiety in adults, including college students (13). Late adolescence and early adulthood represent a key developmental period, and dance-based activities are increasingly embedded in university curricula and extracurricular programmes worldwide (14). However, the evidence for dance training effects on physical fitness, particularly core muscle strength, has not been comprehensively synthesised in this population. This narrative review therefore aims to describe the available evidence on dance training for core muscle strength and physical fitness in higher education settings.

### Core musculature: basic anatomy and function

The core musculature serves as the anatomical and functional centre of the body, providing a stable foundation for all limb movements (15). As outlined in the introduction, understanding core muscle function is essential before examining how dance training may influence it. The core muscles work in coordination to stabilise the spine, transfer forces between the upper and lower extremities, and maintain postural control during both static and dynamic activities (16). The core muscles bridge the upper and lower extremities (17) and contribute to spinal stability and neuromuscular control (18). Enhancement of core muscles is beneficial not only for athletes but for all ages, improving fitness performance and injury prevention (12, 19, 20). A stable, strong core transfers force efficiently from the trunk to the limbs (21). Core training has gained focus for its ability to improve balance, health, lower-extremity strength, core stability, and power transfer (22–24).

**Figure 1:**
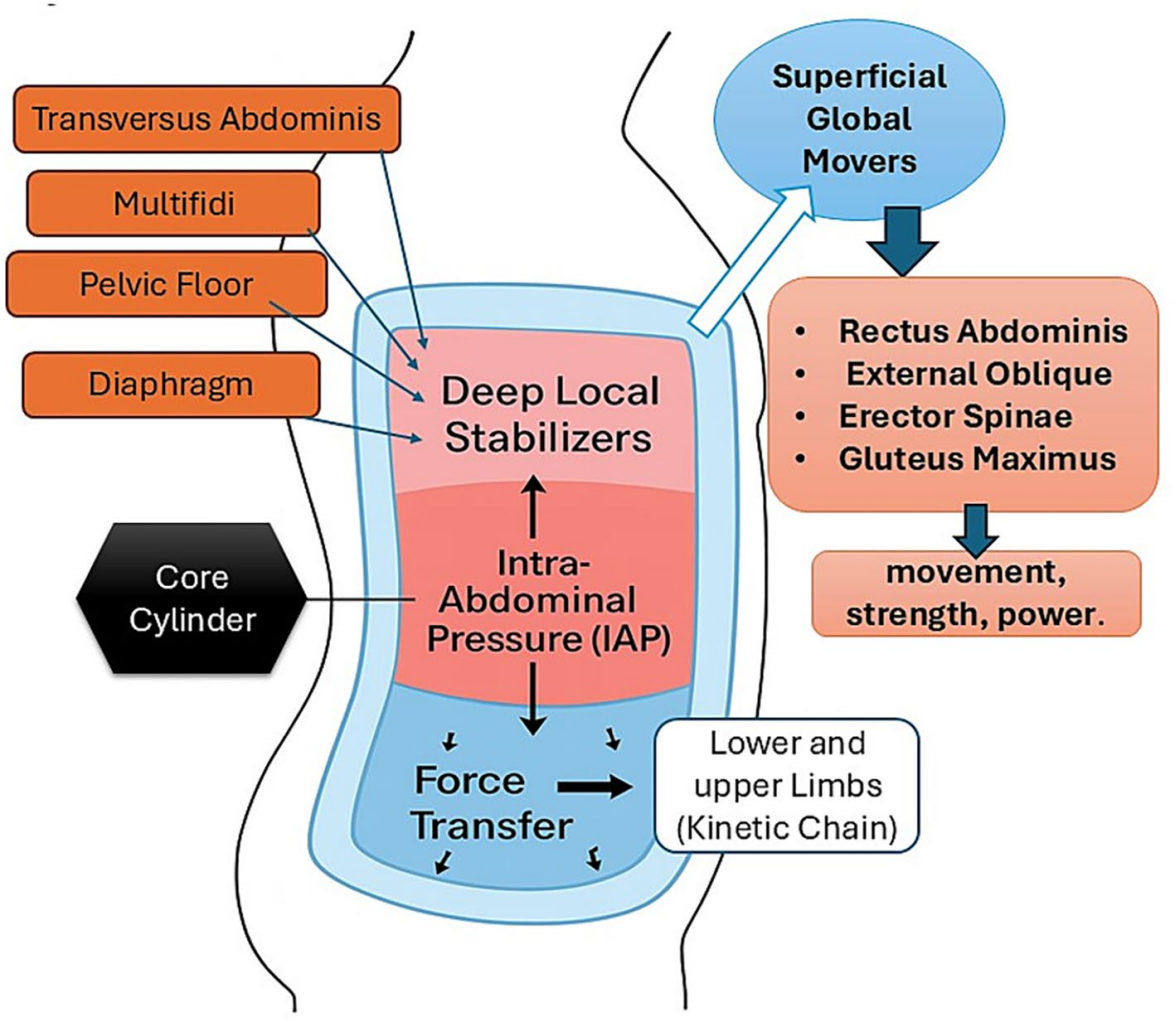
The “muscular box” concept of core stabilisation. The core functions as a rigid cylinder with the abdominals in the front, paraspinals and gluteals in the back, the diaphragm as the roof, and the pelvic floor and hip girdle musculature as the bottom. This configuration provides proximal stability for distal mobility during dance and athletic movements.

Rotational torque around the spine is produced by the activation of the core muscles. Most studies indicate a unique sequencing of timing and intensity of muscle activation, which starts from the opposite side and results in both rotation and force generation (25). They make the trunk or central body stiff, forming a rigid cylinder (26). This “rigid cylinder” concept conceptualizes the core as a muscular box, with the abdominals anteriorly, paraspinals and gluteals posteriorly, the diaphragm as the roof, and the pelvic floor and hip musculature as the base. Together, these structures work in coordination to generate intra-abdominal pressure and provide spinal stability during dynamic movement as shown in Figure 1.

**Figure 2:**
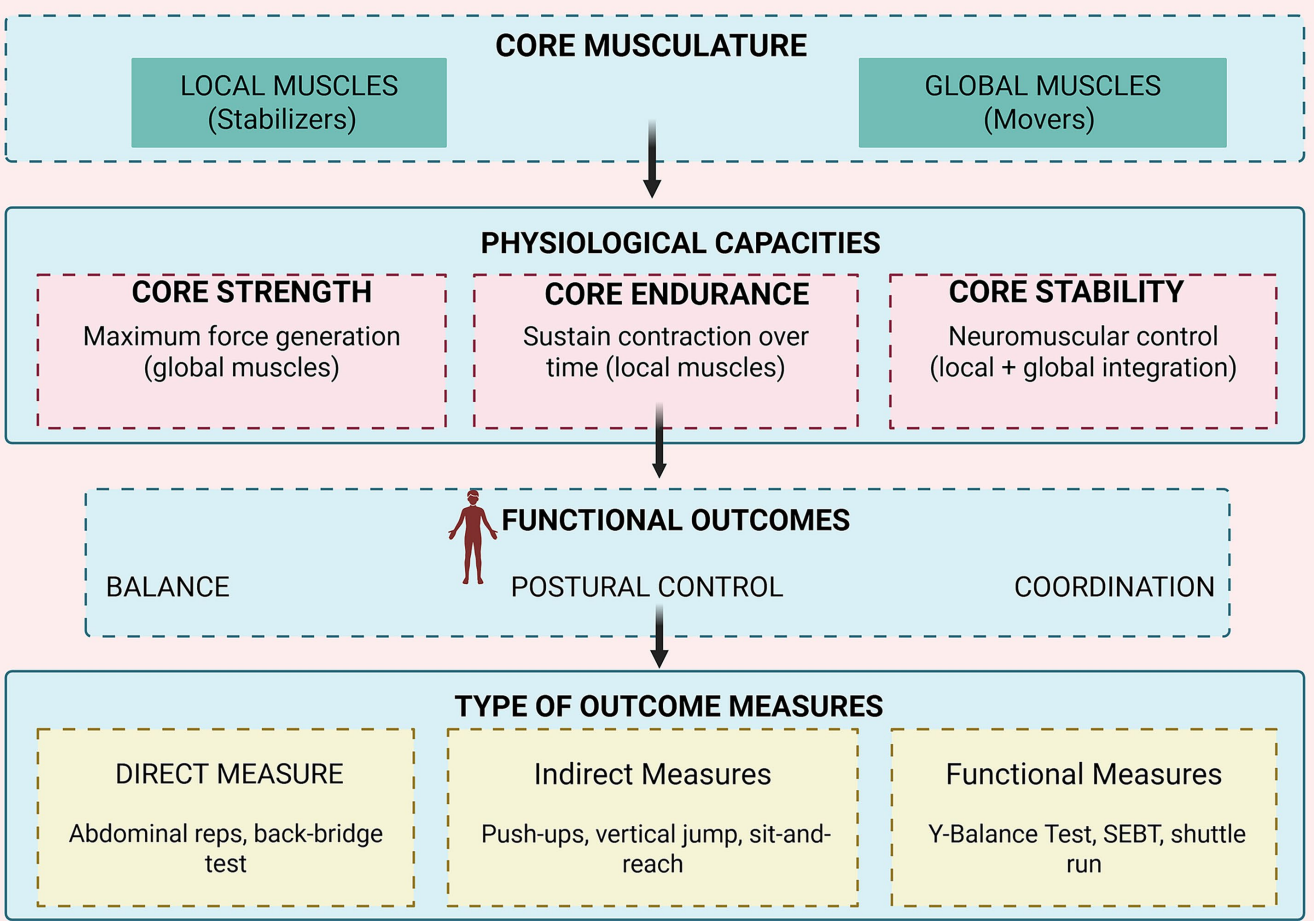
Conceptual framework illustrating the relationship between core musculature, physiological capacities (strength, endurance, stability), functional outcomes (balance, postural control, coordination), and categories of outcome measures used in the reviewed studies.

However, definitions of core and core stability vary widely in the literature, with multiple tests measuring core stability and confusion regarding whether results represent core strength or core endurance (27). Understanding the basic function of core muscles leads to examining how they can be trained. Based on the anatomical understanding of core musculature, it is important to distinguish between related but distinct physiological constructs. Figure 2 presents a conceptual framework showing the pathway from core anatomy to physiological capacities, functional outcomes, and the types of measures used to assess them.

### Core strength training: general principles

Core Strength Training (CST), a novel concept, was initiated in the 1990s for the retraining of athletes (28). This training is widely recognised and appraised in the 21st century. Core Strength Training is acknowledged as a critical factor boosting an athlete’s performance, covering multiple domains resulting in the development of abilities like flexibility, balance, endurance and coordination (29). Core Musculature around the trunk and pelvis is fundamental in maintaining the balance and centre of gravity of the body, and also effective neuromuscular control (30). Core Strength Training is commonly incorporated into musculoskeletal injury prevention programs (31). Balance and core stabilisation exercises refine the physical performance and decrease the incidence of injuries (32). The effect of CST is favourable for the martial arts athletes owing to its benefits, like enhanced centre of gravity balance and muscle performance (33). CST fundamentally incorporates core stability training (for balance, posture, and power in complex movements), joint care training (to protect vulnerable joints from injury) and dedicated core training tailored to individual needs (30). While general core training principles apply to many populations, dance styles place unique demands on core musculature as described below.

### Dance styles and their demands on Core musculature

**Figure 3:**
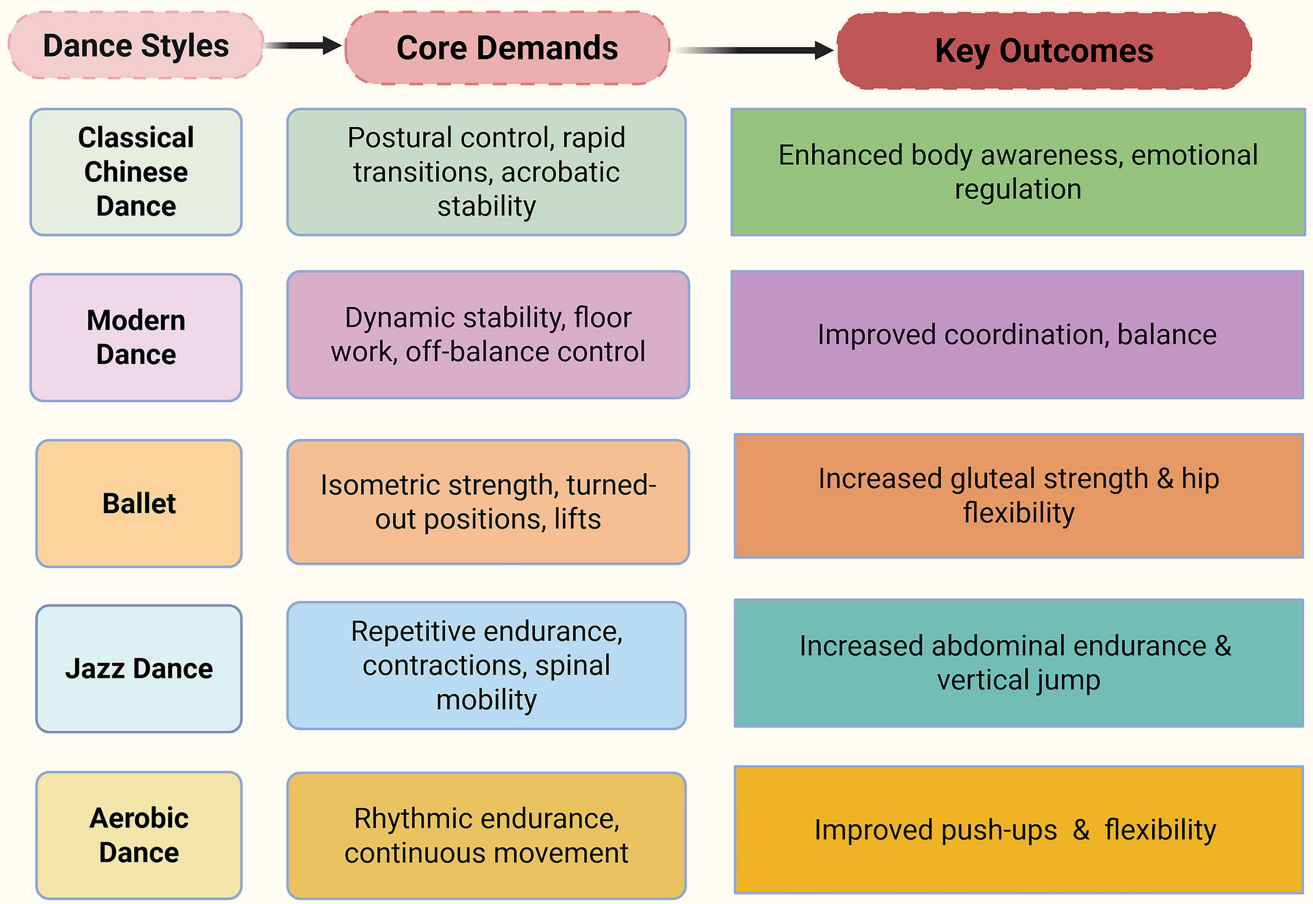
Summary of dance styles, their associated core demands, and key outcomes.

Multiple studies on sports medicine and kinesiology accentuate the importance of core strength training and its advantages. Core strength and stability can be successfully developed through dance styles and training methods, including Classical Chinese dance, modern dance, and ballet (34). Different dance styles place unique demands on core musculature based on their specific technical requirements as shown in Figure 3. The Classical Chinese Dance and modern dance stipulate definite control, smooth flow, and perpetual balance (7, 35, 36).

The Classical Chinese Dance is a combination of multiple aspects involving acrobatics, martial arts, and classical aesthetics, which requires a strong core involvement for stability and control (37, 38). Modern dance has a strong emphasis on the flexible and evocative movements (39). High levels of core stability are required for ballet training in order to perform exact technical moves like lifts, extensions, and turns (40). Beyond the inherent demands of dance techniques, several studies have examined core training effects in various populations relevant to this review.

### Broader evidence for Core training and dance-related outcomes

Some of the evidence-based advantages are:

- A meta-analysis by Ngo et al. (1) of 36 studies concluded that strength and conditioning significantly improves lower body power (*g* = 0.90), upper body strength (*g* = 0.98), and flexibility (*g* = 0.86) in dancers (all *p* < 0.05).
- A meta-analysis by Yang et al. (41) of 15 studies (351 dancers) found that strength training significantly improves muscle strength (ES = 1.84, 95% CI: 0.90–2.77, *p* < 0.001) and muscle power (ES = 0.64, 95% CI: 0.30–0.98, *p* < 0.001) in dancers, although effects on body composition and flexibility were not statistically significant.
- A study examining the effects of a 12-week core strengthening and weight training programme on muscle strength, endurance, and flexibility in male school-aged athletes reveals that young athletes can increase their physical fitness with both weight training and core strengthening (42).
- A study analysing the influence of core training on the physical fitness and athletic performance of high-level aerobic gymnastics athletes. Following the core training intervention, the experimental group’s competitive abilities and physical fitness substantially improved (43).
- A study investigating the impact of abdominal core strength training on the stability of the body in Tae Kwon Do athletes after 3 months of training shows that there is an enhancement of core muscle strength, postural stability, and physical performance (44).
- According to a study of young Chinese dancers, the most often injured areas were the lower back, knees, and ankles, with a 52.5% injury rate after a year (38).
- Along with the physical benefits of basic dance training, it contributes to the psychological and social well-being of the students. Their mental health, emotional regulation, and cognitive function is improved (14, 45). A study on 172 college students in China to see the effect of Classical Chinese Dance on self-awareness and emotional regulation showed that it significantly improves these parameters (46).
- A 16-week core strength training programme using a yoga ball significantly improved core stability, endurance, explosive power, and muscle coordination in undergraduate sport dancers (47).

## Methods

### Study design

This article is a narrative review. A narrative approach was chosen because the available literature is characterised by substantial heterogeneity in dance style, participant characteristics, intervention structure, and outcome measurement, which precludes formal meta-analytic pooling. The study was not pre-registered. As a narrative review, this paper did not perform a formal quality assessment of individual studies (e.g., using the PEDro scale or Cochrane Risk of Bias tool), and selection of studies and synthesis of evidence reflect the author’s judgement.

### Information sources and search strategy

Electronic databases searched were PubMed/MEDLINE, Google Scholar, EBSCO, ScienceDirect, and IEEE Xplore. Searches for eligible empirical studies were conducted between January and June 2025 and covered publications indexed from January 2000 to December 2024. An updated top-up search in May 2025 captured early-2025 publications meeting eligibility. Reference lists of eligible articles and relevant review papers were hand-searched to identify additional studies not captured by electronic search.

Search terms were combined using boolean operators:

(“dance training” OR “dance intervention” OR “dance class” OR “dance conditioning”) AND (“core” OR “trunk” OR “lumbopelvic” OR “abdominal” OR “stability” OR “endurance”) AND (“university” OR “college” OR “undergraduate” OR “higher education” OR “students”)

Searches were limited to peer-reviewed empirical studies published in English involving human participants. Conference abstracts, dissertations, editorials, letters, and study protocols without results were excluded at the screening stage.

### Eligibility criteria

*Inclusion criteria*: Studies were considered eligible if they met all of the following:

- Population: college or university students, or participants clearly recruited within a tertiary education context (e.g., university dance elective, campus-based class).
- Intervention: a structured dance or dance-conditioning programme serving as the primary exposure. Eligible dance styles included, but were not limited to, classical ballet, contemporary dance, modern dance, jazz technique, aerobic dance, Zumba, hip-hop/dancehall, sport dance, and classical Chinese dance.
- Comparator: any comparator was acceptable, including pre–post designs, passive control groups, and active control groups.
- Outcomes: studies were eligible if they reported at least one quantifiable outcome related to core/trunk function or core-related physical fitness. Outcomes were grouped as: (a) direct trunk/core measures, including TrA activation, trunk endurance tests, and abdominal repetition/curl-up tests; (b) composite or task-embedded measures, including core-strength batteries, dance technique/kicking scores, dynamic balance, postural control, proprioception, limb-symmetry indices, and hop-based injury-risk tests; and (c) indirect fitness proxies, including push-ups, hip strength, flexibility, coordination, lower-limb power, balance, and self-reported body awareness. Outcomes were classified according to the actual test used rather than the label assigned by the original study.
- Study design: empirical quantitative studies (randomised controlled trials, non-randomised controlled trials, quasi-experimental designs, cohort studies, or pre–post studies without a control group) published in peer-reviewed journals.

*Exclusion criteria*. Studies were excluded if they:

- Enrolled children, adolescents, or mixed-age samples from which university student data could not be separated.
- Reported outcomes only for non-dance physical activity without a dance component (e.g., pure resistance training, yoga without dance content, Pilates only).
- Were non-empirical in nature (narrative reviews, opinion pieces, editorials, case reports without quantitative outcomes).
- Did not report at least one outcome relevant to core or physical fitness as defined above.

### Study selection

Titles and abstracts identified through database searches were screened independently by the author for relevance to the target population (college/university students) and the intervention (structured dance training). Full texts of potentially eligible papers were then retrieved and assessed against the eligibility criteria. The reasons for exclusion at the full-text stage were recorded. Given this is a narrative review, a PRISMA flow diagram was not formally generated; however, the selection process is described transparently above.

### Data extraction

For each included study, the following information was extracted into a standardised table: author(s) and year; study design; sample size and characteristics (sex, mean age where reported, educational context); dance style and intervention content; intervention duration and session frequency; primary outcome measure(s) relevant to core/trunk function or physical fitness; and direction and magnitude of reported changes (pre/post means ± SD, between-group differences, *p*-values, and effect sizes where available).

### Synthesis approach

Because of the heterogeneity of dance styles, populations, outcome measures, and intervention parameters, findings were synthesised narratively rather than quantitatively. Studies were categorised into three groups: (1) studies reporting direct trunk/core function measures (Table 1, *n* = 3 studies); (2) studies reporting composite, task-embedded, or postural-stability core measures (Table 2, *n* = 4 studies); and (3) studies reporting indirect core-related fitness outcomes (Table 3, *n* = 6 studies). Within each category, findings were grouped by outcome domain and interpreted in the context of the relevant sample characteristics and intervention design. In this review, the following operational definitions are applied. Core strength refers to maximal force production by the trunk musculature, measured by dynamometry, manual muscle testing, or composite strength batteries. Core endurance refers to the ability to sustain submaximal trunk muscle activation over time, measured by isometric hold tests (e.g., prone or lateral plank duration) or repetition tests (e.g., 1-min curl-up). Core stability refers to neuromuscular control of the trunk during dynamic tasks, inferred from balance, postural-control, and anti-rotation tests. Where source studies do not specify, the construct actually measured is reported.

**Table 1:** Summary of the studies reporting direct trunk and core function measures.

Study	Sample	Intervention and duration	Direct core measure	Key finding
Watson et al. (48) *Dance, Balance and Core Muscle Performance Measures Are Improved Following a 9-Week Core Stabilisation Training Programme Among Competitive Collegiate Dancers*	24 female collegiate dancers	9-week core stabilisation programme (3 days/week) + routine dance practise Superimposed on existing training	• TrA activation (ultrasound/ADIM) • Trunk flexor/extensor endurance • Hip abductor strength	TrA activation improved across all 4 instruction conditions (*p* ≤ 0.05); SEBT and single-leg balance (*p* ≤ 0.01); pirouettes 2.21 → 2.55 (*p* = 0.011); all strength measures improved (*p* ≤ 0.05) except heel raise.
Donath et al. (49) *The Effects of Zumba Training on Cardiovascular and Neuromuscular Function in Female College Students*	30 female college students	8-week Zumba (salsa/aerobics-based) (2 days/week; 100% attendance) Instructor-led group sessions	• Trunk endurance: Swiss Global Trunk Strength Test (prone, lat-left, lat-right) • SEBT (balance) • 6-MWT • Stand-and-reach • Jump-and-reach	Trunk endurance and dynamic balance improved significantly; Flexibility and jump performance: NOT significantly improved (*p* > 0.05).
Komeroski et al. (50) *Strength and Flexibility in Beginner Jazz Dancers*	8 novice female university dancers (pre–post, no CG)	12-week jazz technique training (2 sessions/week) Curriculum-based technique class	• 1-min abdominal repetition test • Vertical jump • Handgrip strength • Hip/spinal ROM	Trunk endurance: 28.50 → 33.50 reps (+17.5%). Vertical jump: 33.25 → 37.31 cm (+12.2%). Spinal and hip ROM: all improved. Handgrip: no significant change.

ADIM, abdominal draw-in manoeuvre; CG, control group; 6-MWT, 6-Minute Walk Test; ROM, range of motion; SEBT, Star Excursion Balance Test; TrA, transversus abdominis; *p*, probability value.

**Table 2:** Summary of the studies reporting composite, task-embedded, or postural-stability core measures.

Study	Sample	Intervention and duration	Composite, task-embedded, or postural-stability core measures	Key finding
Kalaycioglu et al. (34) *Effect of a Core Stabilisation Training Programme on Performance of Ballet and Modern Dancers*	24 university ballet and modern dancers	8-week CST (3 days/week, 45–60 min/session) Physiotherapist-supervised (2 days) + independent (1 day)	• Vertical jump • Dynamic balance • Coordination • Proprioception • Hip flexion isokinetic peak torque	Significant improvements in vertical jump, dynamic balance, proprioception, and coordination (*p* < 0.05) post-CST. However, hip flexor isokinetic peak torque decreased significantly (*p* < 0.05).
Yu et al. (12) *Experimental Effects of Multi-Dance Sport Training on Student Performance: A Dual Analysis of Physical Fitness and Aesthetic Skill Development*	90 female PE majors RCT; *n* = 30/group	16-week sport dance training (Rumba vs. Waltz vs. Yoga) Systematic structured sessions	• Core strength (standardized battery) • Vertical jump • Agility • Flexibility	All three groups: significant improvements in core strength, lower-body power, agility, and flexibility (*p* < 0.05).
Yan et al. (51) *The effect of 12-week combined balance and plyometric training on dynamic balance and lower-extremity injury risk in college dancers*	30 female college dancers; BP *n* = 15, PL *n* = 15	12-week balance + plyometric training alongside regular technical dance training; 3 sessions/week; BP included unstable-surface balance exercises	• Dynamic Posture Stability Index • COP displacement • Limb Symmetry Index • single-leg hop-based injury-risk tests	Combined balance–plyometric training improved dynamic balance and lower-limb injury-risk indices versus plyometric training alone, supporting dance-relevant postural control and core-related neuromuscular control.
Peng (65) *Influences of Abdominal Core Strength Training on Sport Dancing*	46 college students majoring in sport dance	4-week abdominal core strength training Added 4 core exercises per class (15 min)	• Dance kicking technique score (4 components) • Equilibrium and stability Test (unipedal stance)	Kicking score — EG vs. CG: kicking total score 89.93 vs. 79.62 (*p* < 0.01); balance: EG held ≥15 s (*n* = 10), CG ≥ 15 s (*n* = 2), *p* < 0.05.

BP, balance–plyometric group; CG, control group; COP, centre of pressure; CST, core stabilisation training; EG, experimental group; PE, physical education; PL, plyometric-only group; RCT, randomised controlled trial; p, probability value.

**Table 3:** Summary of the studies reporting indirect core-related physical fitness outcomes.

Study	Sample	Intervention and duration	Indirect core-related fitness outcomes	Key finding
Kuang et al. (6) *The Effect of Aerobic Dance Programme on Sustained Attention and Physical Fitness of University Students*	60 university women RCT: EG *n* = 30, CG *n* = 30	8-week aerobic dance (3 days/week)	• Push-ups • Sit-and-reach • 800 m run • ANT (sustained attention)	Push-ups: 6.37 → 11.40 reps (+79%). Sit-and-reach: 16.03 → 18.52 cm (+15.5%). Both significantly improved vs. CG.
DiPasquale and Wood (53) The Effect of Classical Ballet and Contemporary Dance Training on Hip Extensor Flexibility and Strength in Novice Dancers: A Pilot Study	29 college students Pilot: EG *n* = 22, CG *n* = 7	11-week ballet + modern dance (160 min/week)	• PSLR hamstring flexibility (goniometry) • Hip extensor/flexor strength (MMT break test)	PSLR: Left 69.82° → 99.77°, Right 75.18° → 101.36° (both *p* = 0.001). Right hip extensor strength significantly increased. Authors link hip extensors to posterior core stabilisation. No direct trunk test administered.
Stošić et al. (52) *Effects of an Exercise Programme on the Coordination and Explosive Power of University Dance Students*	54 female university students	10-week hip-hop + Dancehall dance + trunk and leg muscle strengthening (3×/week, 90 min/session)	• 6 coordination tests • Explosive power (SJ and CMJ by Optojump)	Coordination improved in 5/6 variables (*p* < 0.05–0.01; ES > 0.14). SJ: 20.85 → 23.65 cm (*p* = 0.017, ES = 0.51). CMJ: 22.50 → 25.47 cm (*p* = 0.022, ES = 0.57). Agility: n.s.
Limanskaya et al. (59) *The Coordination Abilities Development in Female Students Based on Dance Exercises*	10 female dance majors (pre–post, 3-semester) Curriculum-based	3 semesters modern jazz (2 h/week)	• Static balance (stork stand, seconds) • Vestibular stability • Shuttle run time • Spatial orientation • Rhythmic control	Static balance: 25.45 → 43.98 s (+73%). Shuttle run: 11.17 → 10.95 s (improved). 5/6 coordination tests significantly improved.
Ling et al. (46) *The Effects of Classical Chinese Dance Movements on Personal Awareness and Emotion Regulation*	172 university students (quasi-experimental)	9-week classical Chinese dance therapy (2×/week, 3 h/session; 18 total hours)	• Introspection Scale • Body Awareness Scale • Emotion Regulation Scale (all validated self-report instruments)	Significant improvements in perceived body control, muscle tone/tension, physical control, and emotion regulation. Enhanced neuromuscular integration and embodied awareness reported. No objective core strength metrics.
Babayığit İrez et al. (66) *Aerobic Dance or Step Dance: Which Exercise Can Increase Balance, Flexibility and Muscle Strength of University Students?*	55 university students (mixed sex) Aerobic *n* = 20, Step *n* = 20, control *n* = 15	12-week aerobic vs. step dance (2 days/week, 60 min/session)	• Sit-and-reach (flexibility) • Static and dynamic balance • Leg dynamometer strength • Body weight • Body fat %	Step-dance group: significant improvements in weight, body fat %, balance, flexibility, and leg strength (*p* ≤ 0.05). Aerobic-dance group: significant improvements in dynamic balance and flexibility only (*p* ≤ 0.05). Control group: no significant changes.

ANT, Attention Network Test; CG, control group; CMJ, countermovement jump; EG, experimental group; ES, effect size; MMT, manual muscle testing; n.s., not statistically significant; p, probability value; PSLR, passive straight leg raise (measured by goniometry); RCT, randomised controlled trial; SJ, squat jump.

### Methodological limitations of the narrative approach

As a narrative review, this paper did not perform a formal quality assessment of individual studies (e.g., using the PEDro scale or Cochrane Risk of Bias tool) and did not conduct quantitative pooling. Selection of studies and synthesis of evidence reflect the author’s judgement, which introduces the possibility of bias. These limitations are acknowledged in the Strengths and Limitations section of the Discussion. Readers should interpret the findings accordingly.

### Summary of included studies

At the collegiate and university level, dance training and conditioning classes are often used to support postural control, trunk endurance, and whole-body coordination. In the literature identified for this review, interventions included Chinese classical dance, modern/contemporary dance, aerobic dance, jazz technique, and ballet-based training. It is important to note that most included studies reported indirect proxies of core function rather than direct trunk strength or endurance measures; only a minority used objective core assessments such as ultrasound imaging or isometric endurance testing. We reviewed the available data, and the analysis is given below according to our findings.

Analysis of different studies with direct core measures is described in Table 1.

Studies reporting composite, task-embedded, or postural-stability core measures are summarized in Table 2.

Studies reporting indirect core-related fitness outcomes are summarized in Table 3.

## Discussion

This narrative review set out to synthesise the available evidence on whether dance-based training may improve core muscle strength and physical fitness in university students. The topic is timely, given rising physical inactivity among young adults and the increasing incorporation of dance into higher education curricula worldwide. The main findings indicate that structured dance training may produce meaningful improvements in trunk endurance, dynamic balance, flexibility, coordination and, in some cases, psychological well-being. However, the evidence base also has important limitations that temper the strength of these conclusions.

### Trunk endurance, activation and stabilisation

A recurring theme across the literature is that dance training, especially when supplemented with core-focused exercises, can enhance the function of trunk muscles. Several controlled trials have reported positive changes in direct measures such as transversus abdominis activation (measured by ultrasound), isometric trunk endurance, and composite core strength batteries (12, 48, 49). These observations are mechanistically plausible: dance movements–particularly those requiring rotational control, single-leg stance, and rapid changes of direction–repeatedly load the deep stabilisers. It is less clear, however, whether all dance styles produce equivalent effects. For example, Zumba training has been shown to improve trunk endurance but not flexibility or jump performance (49), whereas jazz training appears to benefit both abdominal endurance and vertical jump (50). Such differences suggest that the transfer to trunk endurance and stability is style-dependent, a point that curriculum designers should consider.

### Balance, coordination and power

Improvements in balance and coordination are among the most consistently reported outcomes. Static balance, dynamic balance (e.g., Star Excursion Balance Test), and vestibular control all improved across several studies, with intervention durations ranging from 8 weeks to three semesters (49). These gains are not surprising: dance inherently demands precise postural adjustments and weight transfers. More notable is the observation that core stabilisation training (as an adjunct to regular dance practise) appears to accelerate these improvements (48). A recent RCT among 30 college dancers further showed that a 12-week combined balance and plyometric programme produced significantly greater improvements in dynamic balance and lower-extremity injury risk compared to plyometric training alone (51). Explosive power, measured by vertical jump or countermovement jump, also increased in interventions that included jazz, rumba, or hip-hop styles (12, 50, 52). The likely mechanism is improved force transfer through a stiffer, more stable trunk – a concept well established in sports biomechanics but less frequently examined in dance research.

### Flexibility and posterior chain strength

Flexibility gains were reported in most studies that measured them, particularly in hamstring range of motion, gluteal strength, and hip extensor flexibility (53). These changes are relevant to core stability and posterior chain support because the hip extensors and gluteals contribute to the posterior wall of the “core cylinder.” Step dance, ballet, and contemporary dance all produced meaningful improvements, whereas Zumba did not (49). This variability reinforces the idea that dance style matters and that generalisations across styles should be made cautiously.

### Psychological and embodied outcomes

Beyond physical parameters, one study reported that classical Chinese dance therapy improved self-reported body awareness, muscle tone perception, and emotional regulation in a large sample of university students. Although these outcomes are subjective, they align with a growing body of evidence that dance interventions can enhance physical self-esteem, reduce anxiety, and improve cognitive health in adult populations. Such psychological benefits may indirectly support core stability, movement quality and reducing maladaptive tension, but this hypothesis requires further testing (45, 54).

### Implications of dance programs in colleges/Universities

Around the world, 6–10% of non-communicable diseases and roughly 10% of deaths are due to physical inactivity (55, 56). In the US and Europe, physical inactivity is responsible for 1–3% of national health costs (57). Thus, encouraging physical activity through exercises or dance could potentially prolong life expectancy and avoid over five million deaths annually (55). Since college students are still in their formative stages and the coming phase will require a lot of physical exertion, we need to enhance their physical training. In recent years, sports dancing has definitely grown in relevance (58). Enhancing physical literacy, cognitive growth, and socioemotional well-being are the results of incorporating fundamental dance techniques into the academic curriculum (59, 60).

### Curriculum design including core-focused modules

Core modules are part of a well-designed curriculum that lays a solid substructure in dance craft, history, theory, and performance. Courses should include:

- Core-centred Modules engrossed in strengthening the core muscles through Pilates, yoga and proprioceptive exercises.
- Technique and movement proficiency are taught through the extensive training of core dance styles such as ballet, contemporary, and hip-hop.
- Modules focused on injury prevention, balance and control, overall health, and fitness of the students.
- Opportunities for performances that let students use their technical and artistic abilities in a work environment.

Training frequency is critical for cultivating physical tolerance and technical competency. A 6–9-week training programme with 3 sessions per week results in enhancing balance and stability as proven in research (48).

- In China, dance has been formally added to higher education since 1985, highlighted by the setting up of professional training programs in institutions like Wuhan Sports University, Beijing Sport University, and Beijing Dance Academy (61).
- Dance is a statutory part of the curriculum as Health and Physical Education (HPE) in Australia, as dance in physical education promotes participation from students of all genders and ethnic backgrounds and supports inclusive education (62).
- In the USA, as part of the SHAPE America program, dance is incorporated into physical education across many educational institutions (63).

### Strengths and limitations

The studies reviewed have several strengths. Several included randomised controlled designs and some employed direct, objective measures of core function such as ultrasound imaging or isometric trunk endurance tests. The inclusion of diverse dance styles (ballet, jazz, Zumba, hip-hop, sport dance, classical Chinese dance) increases the generalisability of the findings across different university programme contexts (64). At the same time, important limitations must be acknowledged. Sample sizes are generally small, and most participants are female (60–100%), limiting generalisability to male students. Intervention durations vary widely (4 weeks to 3 semesters), and outcome measures are heterogeneous, no standardised core assessment battery has been used across studies. Many studies lack active control groups, making it difficult to isolate the specific effect of dance from general physical activity. Furthermore, most indirect outcome studies rely on proxies such as push-ups or sit-and-reach tests rather than direct core strength measures. Finally, as a narrative review, this paper did not perform a formal quality assessment or meta-analysis, and it is possible that some relevant studies were missed despite a detailed search.

## Conclusion

Structured dance training, particularly when supplemented with core-focused exercises, may improve trunk endurance, dynamic balance, coordination, flexibility, and explosive power in college and university students. Direct improvements in trunk endurance have been observed across ballet-based (48, 50) and Zumba-based (49) interventions; transversus abdominis activation has been documented in one collegiate-dancer cohort (48). Indirect benefits, including hamstring flexibility, gluteal strength, balance, and self-reported body awareness, are more consistently reported across diverse dance styles. Nevertheless, the evidence remains constrained by small, predominantly female samples, heterogeneous outcome measures, and a frequent absence of active control groups. Few studies have employed validated direct measures of core strength or endurance. Although dance training appears a feasible and engaging component of university health promotion, definitive conclusions regarding its specific effects on core muscle strength and injury prevention await well-designed randomised controlled trials that use standardised dance protocols, direct core outcome measures, and adequately powered, diverse samples.
